# Application of a Proposed Multi-Positional Circumferential Arm Liposuction Method and Quantification of its Clinical Efficacy Evaluation

**DOI:** 10.1007/s00266-020-02121-2

**Published:** 2021-02-02

**Authors:** Yunpeng Gu, Ning Kang, Qianwen Lv, Yue Qi, Zhenjun Liu, Weiwei Chen, Xuejian Sun, Haodong Chen, Gui-e Ma, Zuoliang Qi

**Affiliations:** 1 The 15th Department, Plastic Surgery Hospital, Chinese Academy of Medical Sciences and Peking Union Medical College, No. 33, BaDaChu Road, Shijingshan District, Beijing, 100144 China; 2 Research Center of Plastic Surgery Hospital, Chinese Academy of Medical Sciences and Peking Union Medical College, No. 33, BaDaChu Road, Shijingshan District, Beijing, 100144 China; 3 The 16th Department, Plastic Surgery Hospital, Chinese Academy of Medical Sciences and Peking Union Medical College, No. 33, BaDaChu Road, Shijingshan District, Beijing, 100144 China

**Keywords:** Circumferential arm liposuction, Multi-pose, Quantification, Clinical efficacy

## Abstract

**Background:**

Upper arm liposuction mainly focuses on the posterolateral region, which may lead to a lack of harmony between the aspirated and unaspirated areas. In addition, the treatment effect of arm liposuction is often evaluated only by preoperative and postoperative photograph comparison and simple measurement; quantitative research on this topic is still lacking.

**Methods:**

The multi-positional circumferential arm liposuction (MCAL) technique was proposed and applied to a total of 34 females in our hospital from 2017 to 2019. Three-dimensional data of 12 patients before the operation and after 2–3 months were collected and processed by 3D imaging, and the volume reduction rate was evaluated quantitatively.

**Results:**

The MCAL method was successfully applied in the clinic, and its surgical effect was quantitatively studied. The mean follow-up time of 12 patients was (75.2 ±13.1) days, and the postoperative volume was significantly reduced. The postoperative volume of patients with type I, type II and type III decreased by (10.79 ±2.55)%, (17.25 ±3.02)% and (22.76 ±3.51)%, respectively.

**Conclusion:**

Our new MCAL technique was successful, maximizing the esthetic results in upper limb contour refinements in the superficial fascial layer. The clinical efficacy of this proposed MCAL method was evaluated by CT and 3D digital technology, which provided further accuracy in demonstrating its effect on the shape of the arm.

**Level of evidence IV:**

This journal requires that authors assign a level of evidence to each article. For a full description of these Evidence-Based Medicine ratings, please refer to the Table of Contents or the online Instructions to Authors https://www.springer.com.

**Supplementary Information:**

The online version of this article (10.1007/s00266-020-02121-2) contains supplementary material, which is available to authorized users.

## Introduction

 Since its introduction in the 1980s, liposuction has become one of the most frequently performed surgical procedures worldwide [1, 2]. It is commonly believed that deep fat deposits exist only in the posterolateral region of the upper arm, on which most present liposuction operations are exclusively focused [3]. During surgery, the aspiration results cannot be dynamically checked at all times. As a result, the contour of the upper arm is improved only in a single dimension, leading to disharmony of the contour of the upper arm. The concept of circumferential liposuction of the upper arm was first proposed by Gilliland et al. [4, 5] in 1997. They believed that the formation of a circular scar from the superficial layer to the deep layer could directly shorten the diameter of the arm, and a small reduction in radius would lead to a dramatic decrement in cross-sectional area. A more progressive procedure, three-dimensional circumferential liposuction (3D-CL), was reported by Hong et al. [6] in 2012. Using 3D-CL, they successively managed arm obesity and skin laxity in severely obese patients. The shoulder and elbow joints in the upper limbs have great mobility, and the contour of the arms is different when the arms are engaged in different degrees of rotation and muscle tension. Thus, circumferential liposuction at multiple positions is necessary to ensure a natural and smooth postoperative body contour. Inspired by this, we proposed the multi-positional circumferential arm liposuction technique of the upper arm with patients in the supine position, in which surgeries are performed with the joints in various positions, and the skin is under different tensions.

For liposuction of the upper arm, the postoperative change in the subcutaneous fat layer of patients is an important indicator for evaluating the clinical efficacy [7–9]. Currently, the pre and postoperative limb girth and corresponding subcutaneous tissue thickness are often used for preliminary judgment in clinical practice [10]. However, there are large errors associated with this method due to differences between individuals, positions and methods, which decreases the objectivity and accuracy of the results. With the development of CT technology, more accurate representations of the anatomical structures of the upper limbs can be obtained to accurately detect changes in the subcutaneous superficial fascia layer thickness and horizontal diameters and areas [11]. However, owing to the disadvantage of radiation exposure with CT, there are only limited studies of CT for evaluating pre and postoperative soft tissue characteristics in liposuction patients. Additionally, because of differences in the inclusion criteria, surgical methods, CT acquisition and analysis techniques, and operative techniques, previous studies have not reached consistent conclusions. Thus, there is still a lack of relevant studies on objective methods for evaluating the results of arm liposuction. 3D imaging technology can be used to evaluate the shape, contour and symmetry of a limb objectively and accurately and to quantify the volume of a specific limb by presenting 3D images of the body in its natural form. Many studies have proven that 3D imaging technology has good reliability, repeatability and accuracy and that the accuracy continues to increase through continuous improvements in scanning methods and data processing technology [12]. In recent years, 3D imaging technology has been extensively applied in the field of plastic surgery, such as in preoperative evaluation and design, detection of the volume change caused by liposuction, and postoperative morphological evaluation of breast augmentation or breast reduction [13, 14]. Due to its own advantages, 3D technology has great potential for application in clinical and scientific studies of cosmetic surgery for limb contouring.

In general, the primary goal of this work is to propose multi-positional circumferential arm liposuction (MCAL) as a new technique for upper arm liposuction. The MCAL method extends the operation from the posterolateral region of the upper arm to anteromedial and anterolateral regions, axillary, and scapular areas. A total of 34 female patients underwent this technique, including circumferential liposuction in the upper arm, forearm, and whole arm as well as the accessory breast, axillary, and scapula areas. The secondary aim of this study was to evaluate the clinical efficacy of this new method by using 3D imaging technology. The superficial fascial layer of the arm was quantitatively evaluated before and after surgery via CT; the volume change after liposuction was determined by collecting 3D images of patients with different arm contour classifications before and after the surgery; and the possible influence of the classification on the results was analysed.

## Methods

### Patients and Groups

A total of 34 female patients aged between 21 and 38 years (mean 27.3±4.3 years) underwent the MCAL technique. Their body mass index (BMI) ranged from 17.8 to 27.4 kg/m^2^ with an average of 21.8±2.5 kg/m^2^. The mean follow-up period was 5.3±4.3 months. The criteria for the selected patients were as follows: no prior arm fat aspiration procedures; thickness of the subcutaneous tissue detected as measured by the pinch test is no less than 1.5 cm; normal arm movement and sensory function; no other diseases. It should be noted that the skill of the surgeon is important in this technique, and the whole operation was performed by the author.

Moreover, according to previous studies [15] and clinical cognition, patients are divided into three types (I, II and III) according to skin qualities and then further divided into several subtypes based upon different grades of skin laxity (Table 1). The sagging distance was defined as the vertical distance between the brachial sulcus to the lowermost border of pendulous skin, at the position of arm abducted and elbow flexed at 90°. Patients with type IIIc were excluded for sever skin laxity.

**Table 1:** The classification of arm deformity types

Type		Photos	Skin quality	Skin laxity
I	I		Good, firm	Minimal,<5 cm
II	IIa		General, +/-fatty streaks	Minimal,<5 cm
	IIb			Moderate, 5–9 cm
III	IIIa		Poor, extensive fatty streaks	Minimal,<5 cm
	IIIb			Moderate, 5–9 cm
	IIIc			Extensive,>9 cm

The skin quality was evaluated by the skin thickness, dermal and posterior subcutaneous tissue thickness, and the existence of fatty streaks. The skin laxity was measured by the vertical distance between the bicipital groove and the bottom of the hanging skin of the upper arm when the arms were abducted 90°. A distance less than 5 cm was an indication of minimal ptosis, while a distance between 5 cm and 9 cm indicated moderate ptosis, and a distance greater than 9 cm was classified as extensive skin laxity.

### Research Methods

#### Preoperative Marking and Incisions

According to the distribution of fat deposits, patients were marked in the standing position with the arms hanging naturally and at 90° of abduction. Subunits of the arm, including the accessory breast, axilla, lateral thorax and scapular regions were marked as a fan-shaped area apex in the axilla with a radius of approximately 10–15 cm. The obvious intermuscular groove and forearm vasculature were also marked.

**Fig. 1:**
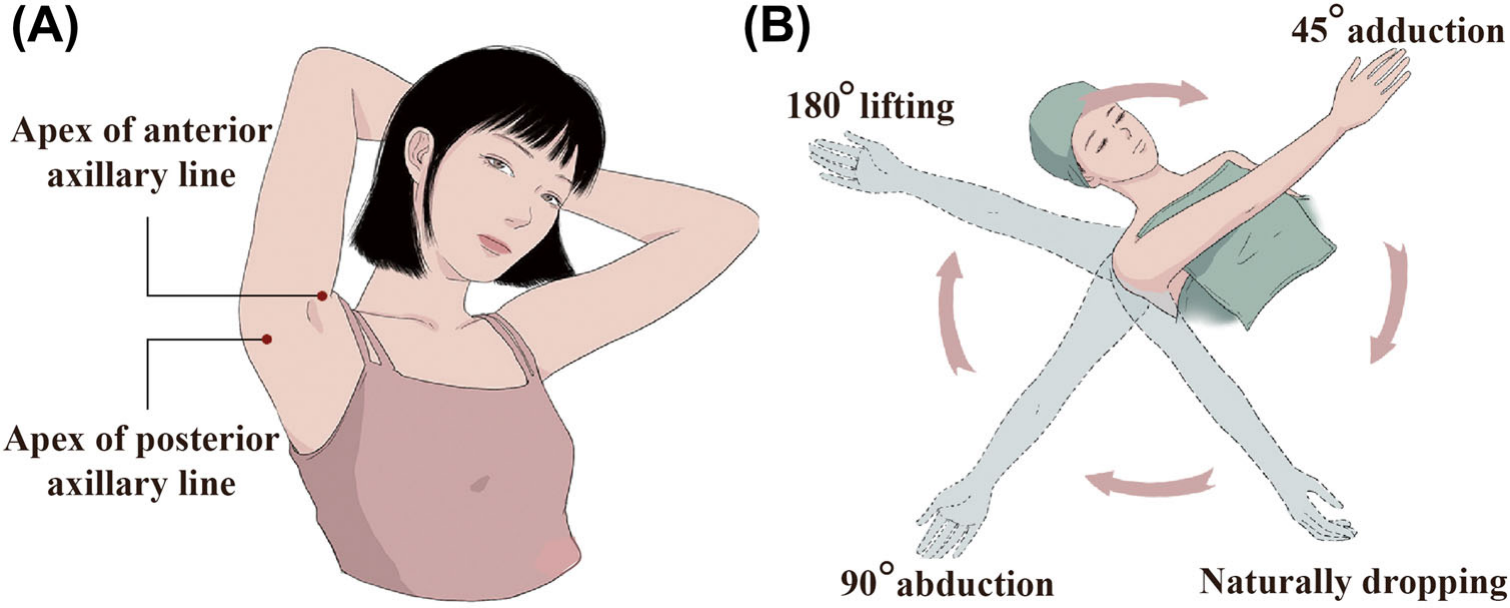
The liposuction pinhole (**a**) and major limb position changes during surgery: 90° abduction position, naturally dropping position, 180° lifting position and 45° adduction position (**b**)

Two 8 mm incisions were made at the apex of the anterior axillary line and posterior axillary line when the shoulder joints were at 90° of abduction and 90° of forward flexion with patients in the supine position, as illustrated in Figure 1a**.** It is important to note that the bilateral access incisions were placed asymmetrically to avoid revealing scars. The final scar lay under the crease of the axilla, such that, with the patient’s arm at the side of the body, the incisions were unnoticeable. The wound protectors were sewn to the incisions to minimize damage to the skin during aspiration.

#### Multi-Positional Circumferential Arm Liposuction

The operation was performed in supine position. After sedation anaesthesia, tumescent solution, consisting of normal saline (1000 ml), 2% lidocaine (20 ml) and 0.1% epinephrine (1 ml), was circumferentially infiltrated into the subcutaneous layer of arm via incisions until wet tumescent. The ratio of infiltrate to fat aspirate was approximately 1-2:1. The maximum total injected lidocaine concentration was 35 mg/kg.

We divided the upper arm and forearm into four regions, namely, anterior, posterior, external and internal regions. During surgery, each arm was placed in the following four different positions as shown in Figure 1b and Figure S1: 90° of abduction, naturally positioned at the side of the body, the 180° lifting position and 45° of adduction. Suction-assisted liposuction (negative pressure approximately700 mmHg) was performed with 3 to 3.5 mm blunt-tip cannulas. The cannula moved through the incisions towards three selected regions of the upper arm in different positions, progressing from the deep layer to the superficial layer of fat tissue to perform fan-shaped cross-tunnelling aspiration. The cannula was manipulated in a back-and-forth motion, which was repeated four to five times. Meanwhile, the contour of the upper arm was checked when muscle groups of the upper arm were contracting and relaxing via the flexion and extension of the elbow joint. The end point of aspiration was determined by observing, touching and grasping the skin of the treated areas. We recommend completely retaining 1-cm-thick tissue under the skin to prevent skin necrosis and significant irregularities. The most common error in the application of the method is injury to the anteromedial vein of the arm due to rough operation.

#### Postoperative Cares and Follow-up

Patients were encouraged to move their arms within 24 h after operation for draining of the tumescent solution. After 1 month postoperatively, patients were allowed to return physical activities as normal. All patients were prescribed to wear compression garments after operation for at least 3 months. The outcomes were assessed during a follow-up visit two to three months postoperatively. Photographs and measurements were taken of all patients for the preoperative and postoperative assessments.

#### Plain CT and Data Processing

Plain CT was performed for 4 volunteers, and their arms were raised to avoid excess radiation. The superficial fascia thickness, circumference and cross-sectional area of the anterior and posterior sides at the maximum plane of the upper arm and forearm were evaluated by CT before surgery and 2–3 months after surgery. All of the obtained structural CT data were preprocessed using the Radida cloud workstation (http://www.radida.com). Soft tissue images were obtained by adjusting the transparency and false colour of the images.

#### 3D Image and Statistical Analysis

**Fig. 2:**
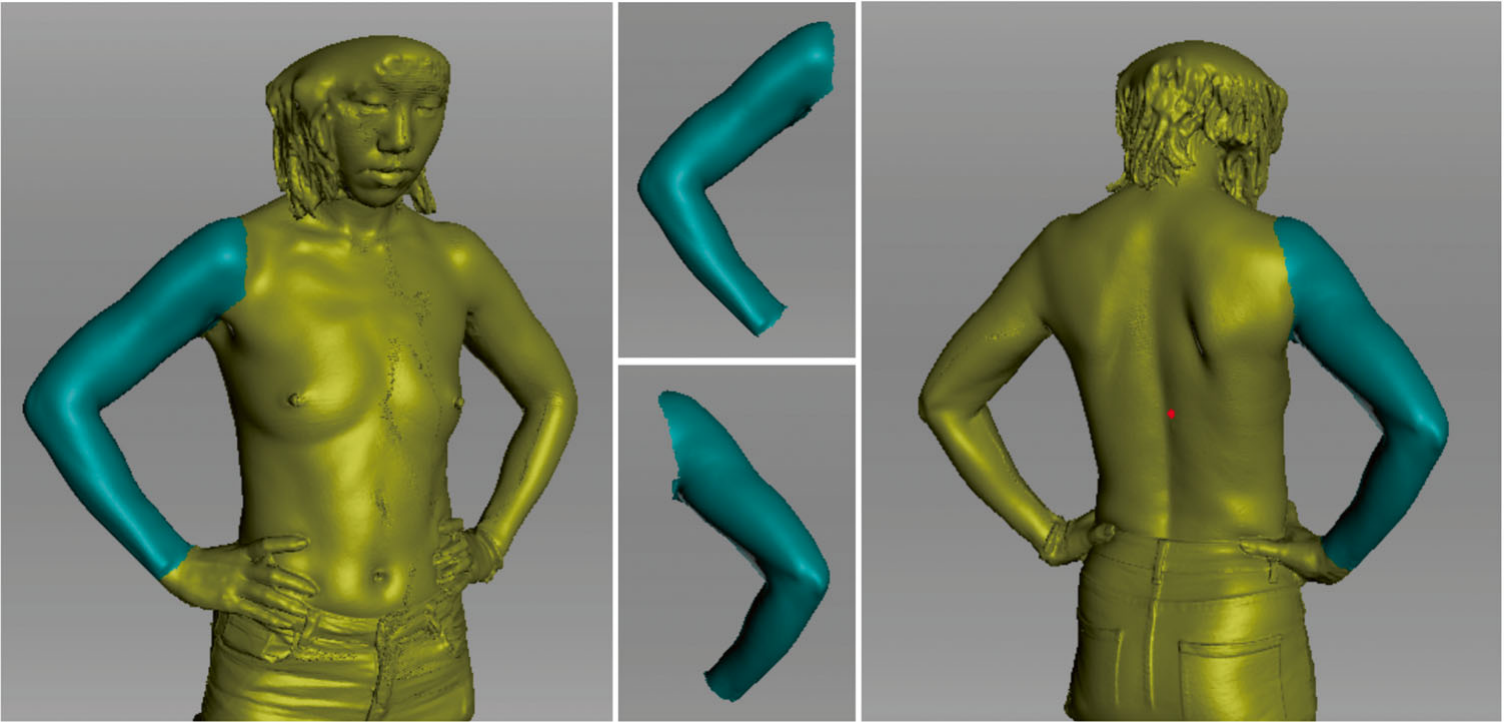
Schematic diagram of 3D data processing for postoperative patients using Artec Studio 12 professional software. Measuring range of the 3D image volumes for each arm (blackish green)

The Artec Eva 3D scanner was used to collect 3D images of the 12 patients before the surgery and 2–3 months after the surgery. Data processing was conducted using the attached Artec Studio 12 professional software (Figure 2).

First, the CT data of 4 patients (8 arms) before and after the surgery and the 3D image volumes of 4 patients (8 arms) for each arm type were analysed by a paired *t*-test to determine the significance of the surgical effect. Next, the volume change in each group was calculated, and the normality of the data in groups I, II and III was tested. Univariate analysis of variance or the rank-sum test was performed to analyse the differences in volume change among the groups and explore the relationship between each arm type and the surgical effect. All statistical analyses were conducted at a significance level of 0.05 (two-sided) using SPSS 17.0.

## Case Reports

### Case 1

#### Type I

This patient was a 27-year-old female with a height of 167 cm, weight of 52 kg and BMI of 18.6 kg/m^2^. The preoperative left upper arm circumference was 27 cm, and the sagging distance was 3.2 cm, while the preoperative right upper arm circumference was 26.5 cm, and the sagging distance was 3 cm. Under sedative anaesthesia, multi-positional circumferential upper arm liposuction combined with liposuction in the accessory breast, underarm and scapular area was performed (Figure S2). A total of 2050 mL of tumescent fluid was injected, and 1600 mL of mixed fluid was aspirated. The amount of pure fat accounted for 800 mL with 400 mL on each side. The three-month postoperative observation showed a straight line on the upper arm and improved skin elasticity. The left upper arm circumference was reduced to 24 cm, and the sagging distance was 2.6 cm, while the right upper arm circumference was 24 cm, and the sagging distance was 2.5 cm.

### Case 2

#### Type IIa

This patient was a 23-year-old female with a height of 161 cm, weight of 48 kg and BMI of 18.5 kg/m^2^. The preoperative left upper arm circumference was 28 cm, and the sagging distance was 3.5 cm, while the preoperative right upper arm circumference was 29.5 cm, and the sagging distance was 3.9 cm. Under sedative anaesthesia, MCAL of the upper arm and 1/3 forearm combined with liposuction in the accessory breast, underarm and scapular area was performed (Figure S3). A total of 2000 mL tumescent fluid was injected, and 1650 mL of mixed fluid was removed. The pure fat content accounted for 970 mL, with 470 mL on the left and 500 mL on the right. The three-month postoperative observation showed that the upper limb was in good shape except for mild scar contracture on the inside of the axilla, and the transition zone between the upper arm and the forearm was natural and smooth. The left upper arm circumference was reduced to 24 cm, and the sagging distance was reduced to 3.1 cm, while the right upper arm circumference was 25 cm, and the sagging distance was reduced to 3.3 cm.

### Case 3

#### Type IIb

This patient was a 22-year-old female with a height of 157 cm, weight of 58 kg and BMI of 23.53 kg/m^2^. The preoperative left upper arm circumference was 31 cm, and the sagging distance was 7.2 cm, while the preoperative right upper arm circumference was 31.5 cm, and the sagging distance was 7.5 cm. Under sedative anaesthesia, MCAL of the upper arm combined with liposuction in the accessory breast, underarm and scapular area was performed (Figure S4). A total of 3000 mL of tumescent fluid was injected, and 2600 mL of mixed fluid was aspirated. The pure fat content accounted for 2300 mL with 1100 mL on the left and 1200 mL on the right. The three-month postoperative observation showed that the posterolateral angular deformity, obesity and sagging of the upper arm were significantly improved, and the upper arm, forearm and body transition zone were natural and smooth. The left upper arm circumference was reduced to 24.5 cm, and the sagging distance was reduced to 3.5 cm, while the right upper arm circumference was 24.5 cm, and the sagging distance was 3.6 cm.

### Case 4

#### Type IIIa

This patient was a 29-year-old female with a height of 165 cm, weight of 59 kg and BMI of 21.67 kg/m^2^. The preoperative left upper arm circumference was 31 cm, and the sagging distance was 4.8 cm, while the preoperative right upper arm circumference was 31 cm, and the sagging distance was 4.9 cm. Under sedative anaesthesia, MCAL of the upper arm combined with liposuction in the accessory breast, underarm and scapular area was performed (Figure S5). A total of 3000 mL of tumescent fluid was injected, and 2600 mL of mixed fluid was aspirated. The pure fat content accounted for 1800 mL, with 900 mL on the left and 900 mL on the right. The three-month postoperative observation showed that the appearance was significantly improved, and the upper arm, forearm and body transition zone were natural and smooth with good skin contraction. The left upper arm circumference was reduced to 25 cm, and the sagging distance was reduced to 3.2 cm, while the right upper arm circumference was 25 cm, and the sagging distance was 3.4 cm.

### Case 5

#### Type IIIb

This patient was a 24-year-old female with a height of 162 cm, weight of 72 kg and BMI of 27.43 kg/m^2^. The preoperative left upper arm circumference was 35 cm, and the sagging distance was 8 cm, while the preoperative right upper arm circumference was 35 cm, and the sagging distance was 8.5 cm. Under sedative anaesthesia, MCAL of the full arm combined with liposuction in the accessory breast, underarm and scapular area was performed (Figure S6). A total of 3500 mL of tumescent fluid was injected, and 3400 mL of mixed fluid was aspirated. The pure fat content accounted for 2350 mL with 1150 mL on the left and 1200 mL on the right. The three-month postoperative observation showed that the appearance was significantly improved, and the upper arm, forearm and body transition zone were natural and smooth. The left upper arm circumference was reduced to 27.5 cm and the sagging distance to 5.1 cm, while the right upper arm circumference was 27.5 cm and the sagging distance was 5 cm.

## Results

### Application of MCAL

Among the 34 patients who accepted the treatment of MCAL, 20 patients underwent circumferential liposuction of the upper arm, 10 patients underwent circumferential liposuction of the upper arm and 1/3 of the forearm, and the other 4 patients underwent liposuction of the whole arm. The average volume of fat removal was 1407.1±455.7 ml. The follow-up period ranged from 2 to 12 months. The activity of the arms was reported to be normal after surgery for all patients. There were no major complications, including hematoma, seroma, contour irregularity, asymmetry of the two arms, skin necrosis, or embolism. Unilateral axillary lymphedema occurred in only one patient one week after the operation and was fully healed after three weeks.

As shown in the case reports, Figure S2–S6 present the preoperative and postoperative photographs of the selected patients. For all patients, the contour of the upper arm was dramatically improved. The “batwing” deformity in the posterior region disappeared, and an inferior straight line appeared on the upper arms. The upper arms, forearms and shoulders were smoothly and naturally connected. No significant postoperative laxity was observed in patients with type IIIa or IIIb, the postoperative skin contraction was quite good, and the degree of relaxation was dramatically improved, especially in patients with type IIb and IIIb.

According to the statistical analysis summarized in Table 2**,** the thickness of subcutaneous tissue in the posterior region decreased by (56.6±6.2)%, while that of subcutaneous tissue in the anterior region decreased by (44.7±9.6)%. Moreover, the average maximum circumference of the upper arm decreased by (16.2±4.0)% and the distance of skin laxity decreased by (29.5±8.9)% after surgery. These results indicated that the fat deposits in both posterior and anterior regions of the upper arm were significantly removed.

**Table 2:** The statistical analysis of fat deposits

Measurements	Change ± SD (cm)	Percentage change ± SD	*t-value*	*p-value*
Thickness of the subcutaneous tissue in the posterior region	2.26 ± 0.83	56.6 ± 6.2%	6.973	<0.001
Thickness of the subcutaneous tissue in the anterior region	1.66 ± 0.58	44.7 ± 9.6%	2.249	0.028
Maximum circumference of the arm	4.91 ± 1.70	16.2 ± 4.0%	24.047	<0.001
Distance of skin laxity	1.53 ± 0.79	29.5 ± 8.9%	8.848	<0.001

The change in thickness of the subcutaneous tissue in the posterior and anterior regions of the upper arm, maximum circumference of the arm and distance of skin laxity after surgery for the 34 patients who underwent the MACL technique. Standard deviations (SD) are also shown. A *p-value* of less than 0.05 means statistically significant.

### Evaluation of the Clinical Efficacy of MCAL

**Fig. 3:**
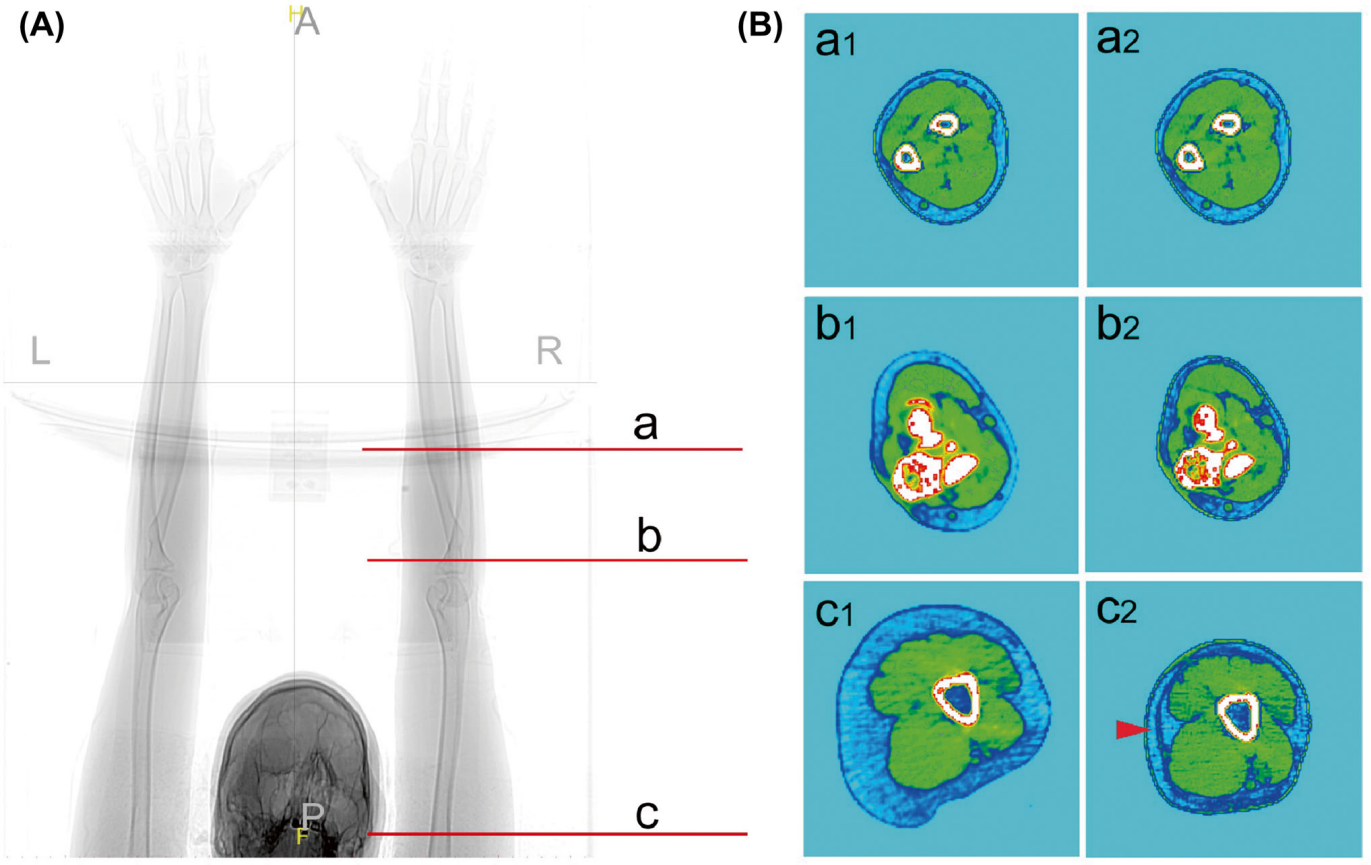
Comparison of pre and postoperative arm CT cross sections. Position selection and observation planes of plain CT scans of volunteers **(a)**. Preoperative (B, a1–c1) and postoperative (B, a2–c2) images of different sections. Circular postoperative scar on the superficial fascia of the upper arm (red arrow in B, c2)

CT was completed with the arms in a raised posture, and the maximum forearm plane elbow joint plane and maximum upper arm plane were taken for pre and postoperative comparison (Fig. 3). Noticeably, circular postoperative scar was found in the superficial fascia of the upper arm (red arrow in Fig. 3B, c2).

**Fig. 4:**
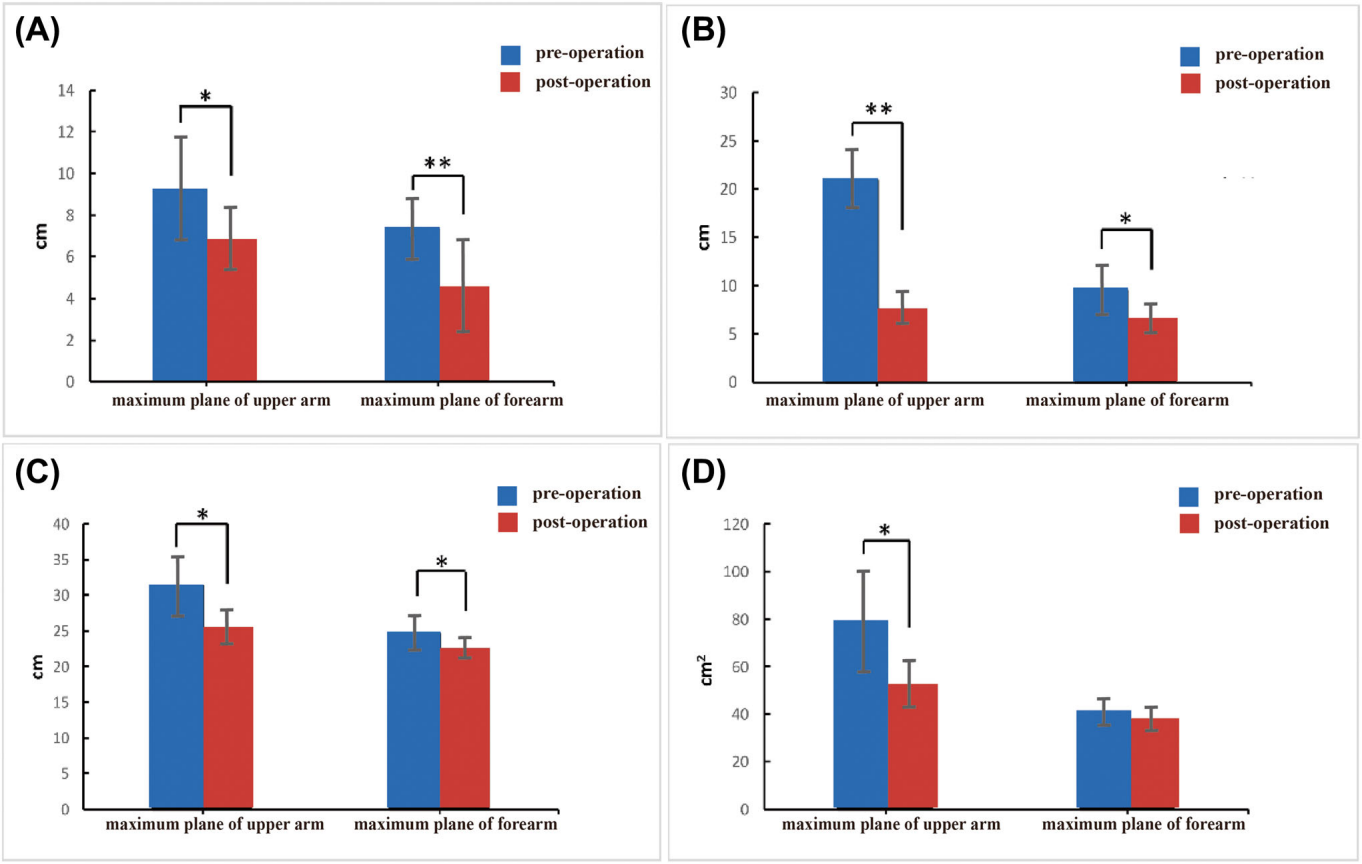
The changes in the fat thickness of the anterior **(a)** and posterior sides **(b)** of the maximum plane of the upper arm and forearm after the surgery; the changes in the circumference **(c)** and cross-sectional area **(d)** of the upper arm and forearm after the surgery. One asterisk stands for *p* < 0.05, and two asterisks stand for *p* < 0.01

As shown in Fig. 4, the fat thickness, circumference and cross-sectional area of the anterior and posterior sides of the maximum plane of the upper arm and forearm changed greatly after the surgery. No significant difference was found in the cross-sectional area of the forearm, but all other data were significantly reduced. Specifically, the posterior fat layer thickness of the maximum plane of the upper arm decreased from (20.8±7.6) mm to (7.6±1.8) mm, with an average reduction of (63.83 ±3.97)%; the anterior fat layer thickness of the maximum plane of the forearm dropped from (7.3±2.2) mm to (4.6±2.4) mm, with an average reduction of (40.0±12.1)% and with p<0.001, indicating a significant difference after the surgery. In addition, the maximum circumference of the upper arm decreased from (31.1±4.3) cm to (25.5±2.5) cm, with a significant reduction of (17.68 ±4.84)%, and its cross-sectional area decreased from (78.6±21.7) cm^2^ to (52.6±9.9) cm^2^, with a significant reduction of (31.55 ±9.39)%. In addition, the maximum circumference of the forearm dropped from (24.5±2.6) cm to (22.6±1.6) cm, with a significant reduction of (7.13 ±4.91)%, and its cross-sectional area dropped from (47.5±10.6) cm^2^ to (40.8±6.7) cm^2^, with a reduction of (12.9 ±7.2)%, but this difference was not statistically significant.

**Fig. 5:**
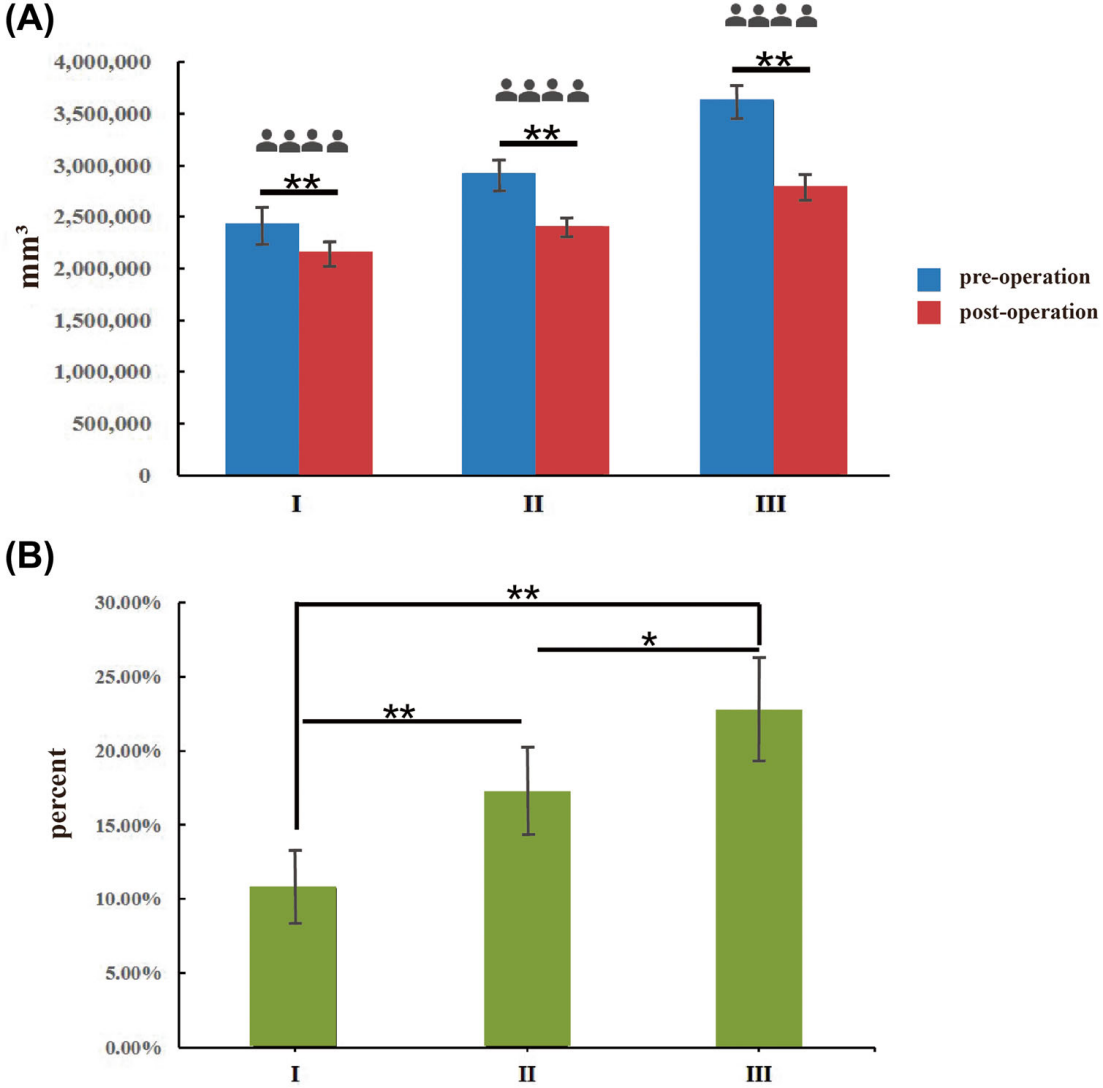
Changes in the upper limb volume of patients with types I, II and III after multi-positional circumferential arm liposuction. **a**: the volume change; **b**: the rate of volume change. One asterisk stands for *p* < 0.05, and two asterisks stand for *p* < 0.01

Based on the results of the 3D imaging analysis, the upper limb volume of the patients with arm types I, II and III changed significantly after MCAL, as shown in Figure 5, with a decrease in (263642±76059) mm^3^, (502792±104230) mm^3^ and (823661±151085) mm^3^, respectively, and the volume was correspondingly reduced by (10.79±2.55)%, (17.25±3.02)%, and (22.76±3.51)%. The results of the statistical analysis indicated that there was a significant difference between arm types II and III in terms of the volume reduction, while there was an even more significant difference between arm types I and II/III in terms of the volume reduction, indicating that the operation resulted in more obvious changes in the upper limb contour of patients with arm types II and III.

## Discussion

Many Asian women with a low BMI also have the need for upper arm liposuction. These patients are usually thin and have tight skin, with almost no obvious laxity, and a thin fat layer. Most of these patients belong to the type I arm classification, which requires a greater surgical effect, and arm liposuction is more difficult in these cases. The results show that the volume change was still statistically significant, indicating that patients with type I arms can also be indicated for circumferential arm liposuction and obtain satisfactory clinical results through surgery. For optimal effect, liposuction is designed to target subcutaneous tissue with a thickness of no less than 1.5 cm [16–18]. However, all liposuction areas, especially the anteromedial side of the upper arm, should maintain at least 1 cm of superficial fat.

Among many imaging measurement and analysis methods, CT and 3D digital imaging technologies show high repeatability and accuracy. CT not only enables volume measurement through 3D reconstruction but also has obvious advantages in the analysis of the internal anatomical structures of the upper limbs. Moreover, it can be used to observe changes in blood vessels and other microstructures as well as quantitatively analyse postoperative changes in the fascia layer. However, CT has the obvious disadvantage of radiation exposure, leading to poor compliance among patients [19]. 3D imaging technology can be used to simultaneously quantitatively evaluate the shape, volume and symmetry of bilateral upper limbs and is conducive to repeated follow-up measurements; it is simple and non-invasive and has high compliance among patients [11]. Therefore, these two methods were adopted in this study for a retrospective analysis of cases. The CT data demonstrated that the fat thickness, circumference and cross-sectional area of the upper arm were significantly reduced after the MCAL. The 3D scan data showed that the MCAL was able to significantly reduce the upper limb volume for patients with three different skin laxity types (type I, II and III). The CT and 3D scan data provided accurate quantitative evaluation of the efficacy of MCAL.

Traditionally, liposuction of the arm was performed almost exclusively on the posterolateral region of the upper arm with less effect of skin retraction [3]. In 1997, Gilliland et al. firstly proposed the concept of circumferential liposuction of the upper arm and revealed its skin retraction effect [4, 5]. They believed that the formation of a circular scar from the superficial layer to the deep layer after circumferential liposuction could directly shorten the diameter of the arm and lead to a dramatic decrement in cross-sectional area. In our study, we reported an imaging evidence of circular scars in the superficial fascia layer after circumferential arm liposuction via CT examination for the first time, which provided a further explanation to the skin-tightening effect of circumferential liposuction. More notably, in our study, there was no supplemental use of brachioplasty or other skin-tightening techniques, such as radiofrequency assistance. Our results showed that sagging of the upper arm was significantly improved after MCAL for patients with mild and moderate skin laxity as well. Satisfactory clinical effects could be obtained by MCAL alone for patients with mild to moderate laxity.

Most operative limbs exhibit edema for 1–2 weeks after circumferential arm liposuction. Fading of edema and scar contraction, hyperplasia and softening are part of the evolutionary process of the change in limb volume after fat aspiration, which is closely related to the doctor’s technique, the degree of trauma, the recovery stage and the individual’s constitution. To avoid confounding effects, all patients were operated on independently by the author, and data were collected 2–3 months after the operation, i.e. when the effect was basically stable. Eventually, the 3D imaging data of 12 patients (4 cases/arm type) and CT data of 4 patients were collected in this study. Despite the small sample size, the total number of samples in the groups could meet the requirements for statistical analysis, and more long-term stable data can be provided to further improve the study over a longer follow-up period.

## Conclusion

We successfully proposed a new MCAL technique that maximized the esthetic results in upper limb contour refinements. This method has been widely applied among patients with different arm types. The clinical efficacy of this proposed MCAL method was evaluated by CT and 3D digital technology. According to the research results, circumferential arm liposuction can significantly improve patients with arm types I/II/IIIa and IIIb, especially those with arm types II/III and loose skin. This study also provided important data supporting the innovative surgical method to further optimize the preoperative evaluation, promote doctor-patient communication, create rational surgical expectations, and enhance patient satisfaction.

## Supplementary Information

Below is the link to the electronic supplementary material.

Supplementary file1 (DOCX 2922 kb)

## References

[1] Duncan DI (2012). Improving outcomes in upper arm liposuction: adding radiofrequency-assisted liposuction to induce skin contraction. Aesthet Surg J.

[2] Matarasso A, Levine SM (2013). Evidence-based medicine: liposuction. Plast Reconstr Surg.

[3] Lillis PJ (1999). Liposuction of the arms. Dermatol Clin.

[4] Gilliland MD, Lyos AT (1997). CAST liposuction: an alternative to brachioplasty. Aesthet Plast Surg.

[5] Gilliland MD, Lyos AT (1997). CAST liposuction of the arm improves aesthetic results. Aesthet Plast Surg.

[6] Hong YG, Sim HB, Lee MY (2012). Three-dimensional circumferential liposuction of the overweight or obese upper arm. Aesthet Plast Surg.

[7] Motley R, Field LM (2008). Morbidity from liposuction under general anaesthesia–‘the elephant in the room’. J R Soc Med.

[8] Garibyan L, Sipprell WH, Jalian HR, Sakamoto FH, Avram M, Anderson RR (2014). Three-dimensional volumetric quantification of fat loss following cryolipolysis. Lasers Surg Med.

[9] Carruthers JD, Humphrey S, Rivers JK (2017). Cryolipolysis for reduction of arm fat: safety and efficacy of a prototype coolcup applicator with flat contour. Dermatol Surg.

[10] Leclere FM, Alcolea JM, Vogt PM (2016). Laser-assisted lipolysis for arm contouring in teimourian grades III and IV: a prospective study involving 22 patients. Plast Surg (Oakv).

[11] Tong Y, Udupa JK, Torigian DA (2017). Chest fat quantification via CT based on standardized anatomy space in adult lung transplant candidates. PLoS ONE.

[12] Wu RW, Yang XN, Jin XL (2018). Three-dimensional volumetric analysis of 3 fat-processing techniques for facial fat grafting: a randomized clinical trial. JAMA Facial Plast Surg.

[13] Wang YR, Sun JJ, Ji K (2015). Percentage volume maintenance in autologous fat graft breast augmentation. Chin J Med Aesth & Cosmet.

[14] Liu CJ, Ji K, Sun JJ (2014). Does respiration influence breast volumetric change measurement with the three-dimensional scanning technique?. Aesthet Plast Surg.

[15] Deng M, Gu Y, Lei H, Liu Z, Ma G (2014). Application of adjacent aesthetic unit combined liposuction in upper arm. Chin J Plast Surg.

[16] Lv Q, Li X, Qi Y, Gu Y, Liu Z, Ma GE (2020). Volume retention after facial fat grafting and relevant factors: a systematic review and meta-analysis. Aesthet Plast Surg.

[17] Kim DH, Byun IH, Lee WJ, Rah DK, Kim JY, Lee DW (2016). Surgical management of gynecomastia: subcutaneous mastectomy and liposuction. Aesthet Plast Surg.

[18] Chia CT, Theodorou SJ, Hoyos AE, Pitman GH (2015). Radiofrequency-assisted liposuction compared with aggressive superficial, subdermal liposuction of the arms: a bilateral quantitative comparison. Plast Reconstr Surg Glob Open.

[19] Herlin C, Chica-Rosa A, Subsol G (2015). Three-dimensional study of the skin/subcutaneous complex using in vivo whole body 3T MRI: review of the literature and confirmation of a generic pattern of organization. Surg Radiol Anat.

