# Supplementary material for: Application of a Proposed Multi-Positional Circumferential Arm Liposuction Method and Quantification of its Clinical Efficacy Evaluation
Source: Aesthetic Plast Surg. 2021 Feb 2;45(3):1115–24. doi: 10.1007/s00266-020-02121-2 (PMC8144089; doi:10.1007/s00266-020-02121-2)
Supplement: Supplementary file 1 — Supplementary file1 (DOCX 2922 kb) [file 266_2020_2121_MOESM1_ESM.docx]

Supporting Information


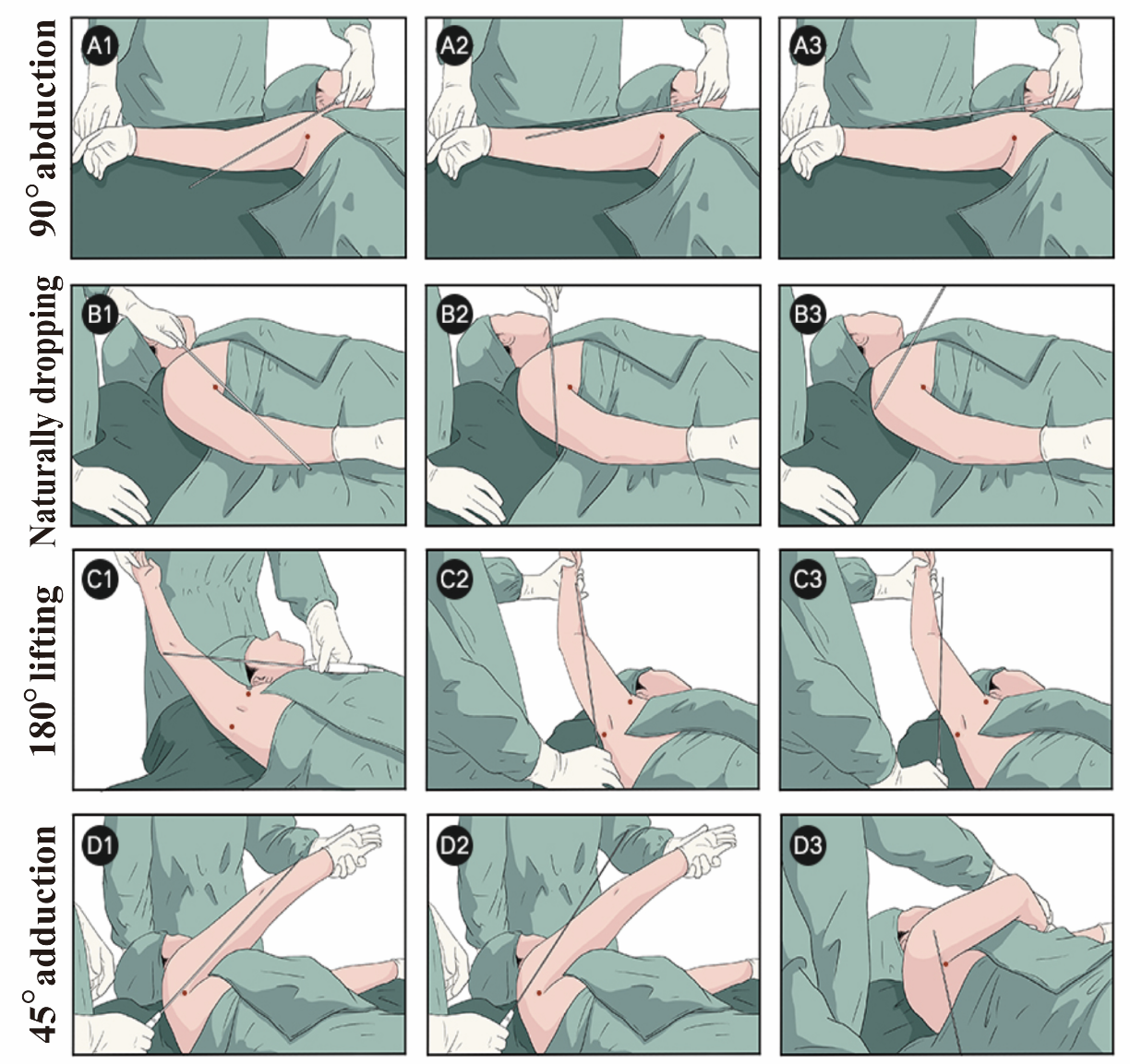


Figure S1. The detailed limb position changes during surgery.

A1 ~ A3: 90° abduction position. The adipose tissue of the anterior, external and internal regions as well as the accessory breast area is aspirated through the anterior axillary needle eye. It should be noted that liposuction in the anteromedial region through the incision at the anterior axillary line is necessary, since the anteromedial region of the upper arm has less fat than other regions, and repeated needle or suction too shallow would lead to uneven or damage to the subdermal structure resulting in mottled hyperpigmentation and skin necrosis.

B1 ~ B3: naturally dropping position. The adipose tissue of the external, anterior and internal regions is aspirated through the anterior axillary needle eye. For deltoid area, a thin arc liposuction needle is required to avoid suction too shallow, causing uneven.

C1 ~ C3: 180° lifting position. The adipose tissue of the posterior, external and internal regions as well as the axilla region is aspirated through the anterior and posterior axillary needle eyes, and the posterolateral fat of the arm and the axilla fat were sucked from far and near.

D1 ~ D3: 45° adduction position. The adipose tissue of the external, posterior and internal regions as well as the scapular area is aspirated through the posterior axillary needle eyes. The elbow, which is almost without fat deposits and rich in fibrous membrane-like tissue, should not be suctioned due to the high potential for contour deformities.


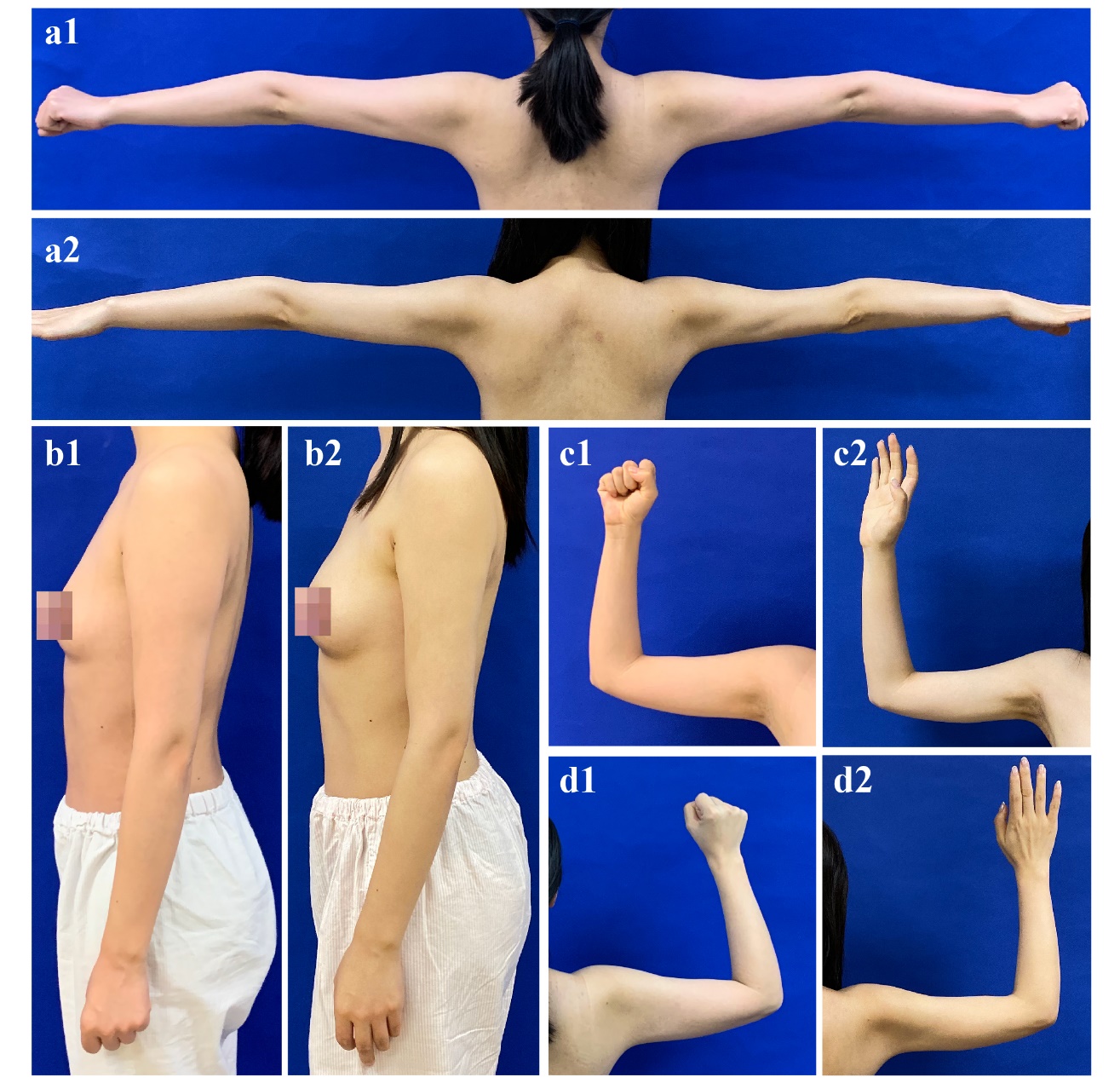


**F****igure S2.** The comparison of a type I patient between pre-operation (a1, b1, c1, d1) and three-month post-operation (a2, b2, c2, d2).


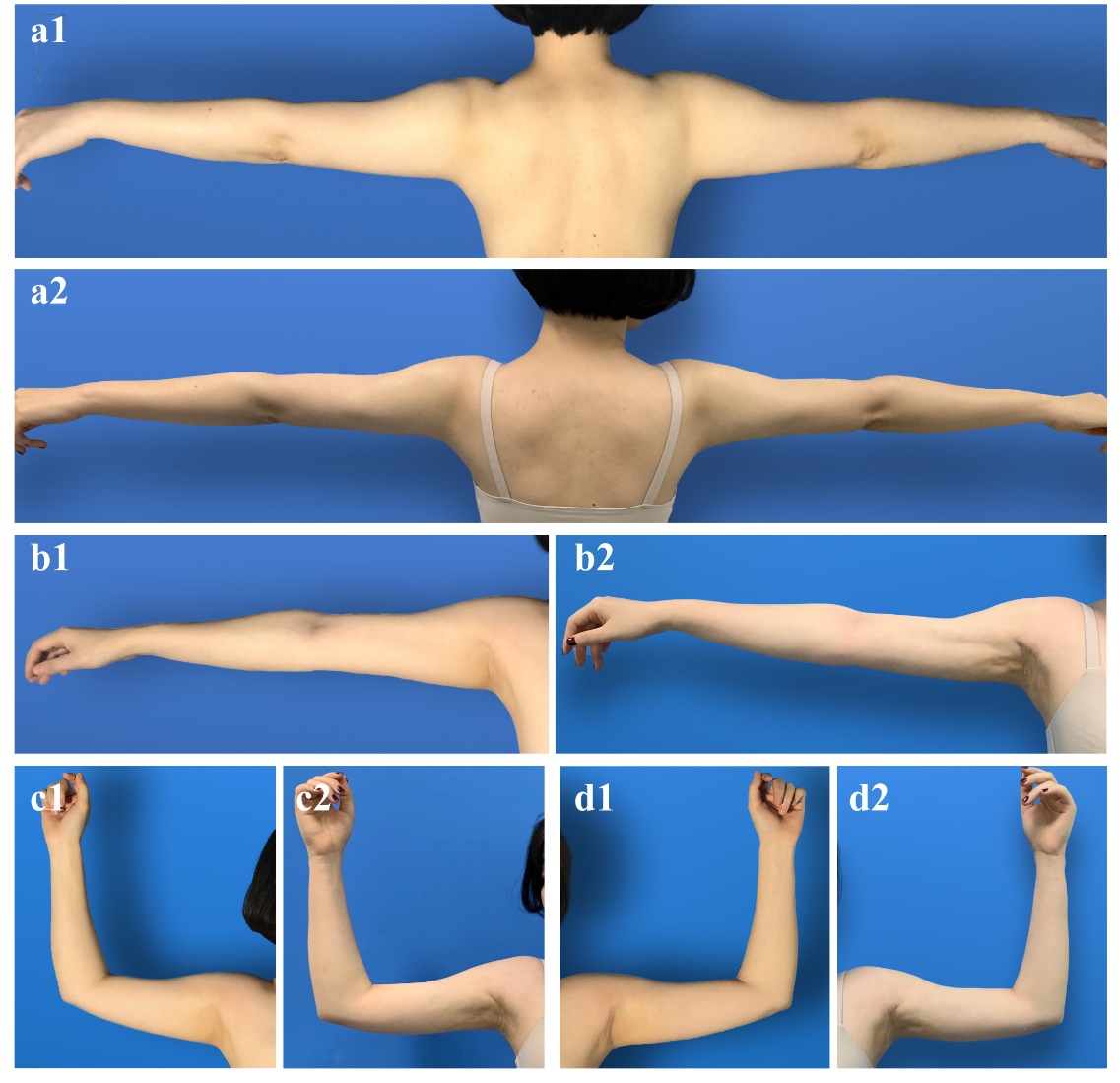


**Figure S3**. The comparison of a type IIa patient between pre-operation (a1, b1, c1, d1) and three-month post-operation (a2, b2, c2, d2).


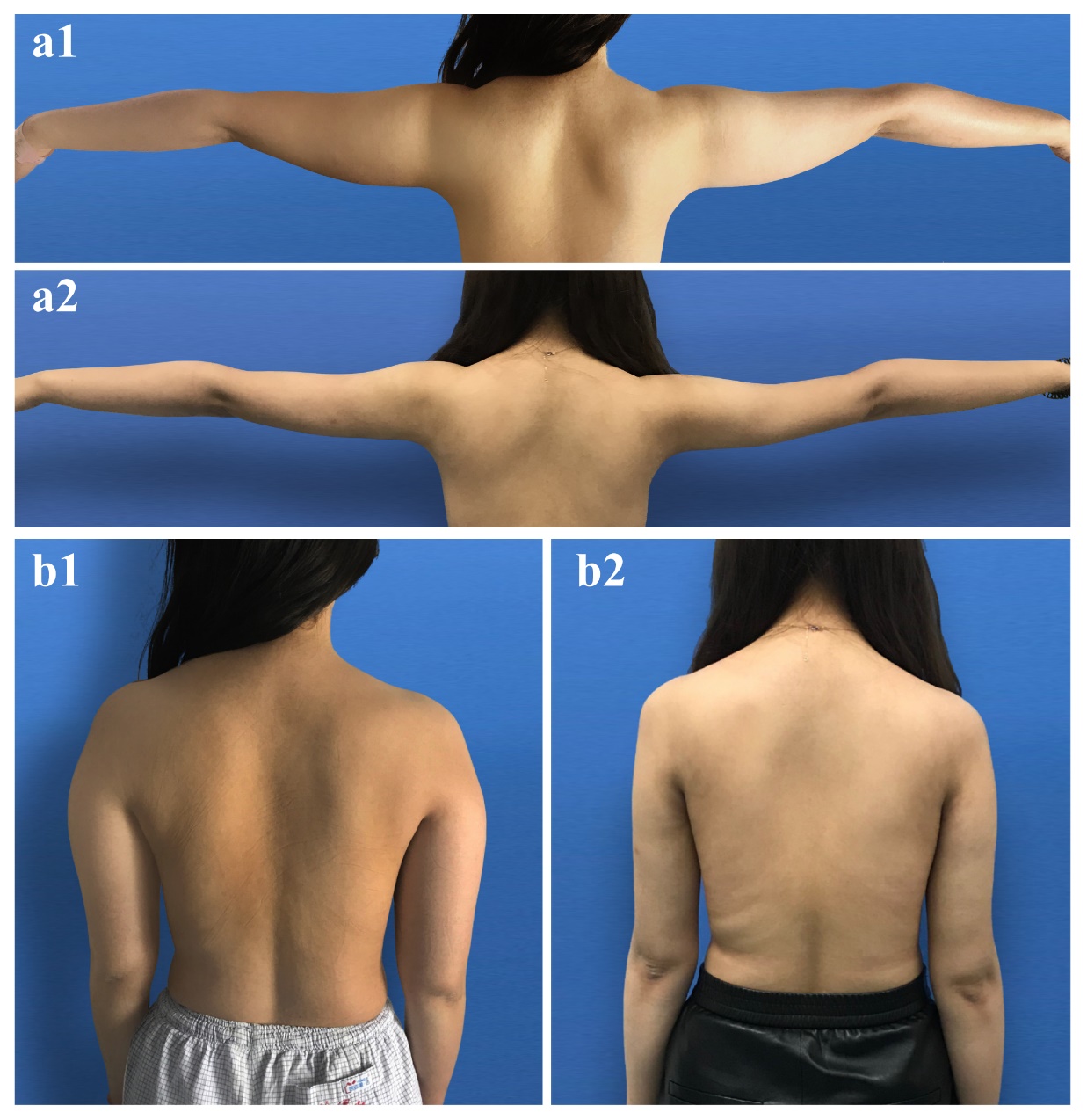


**Figure S4**. The comparison of a type IIb patient between pre-operation (a1, b1) and three-month post-operation (a2, b2).


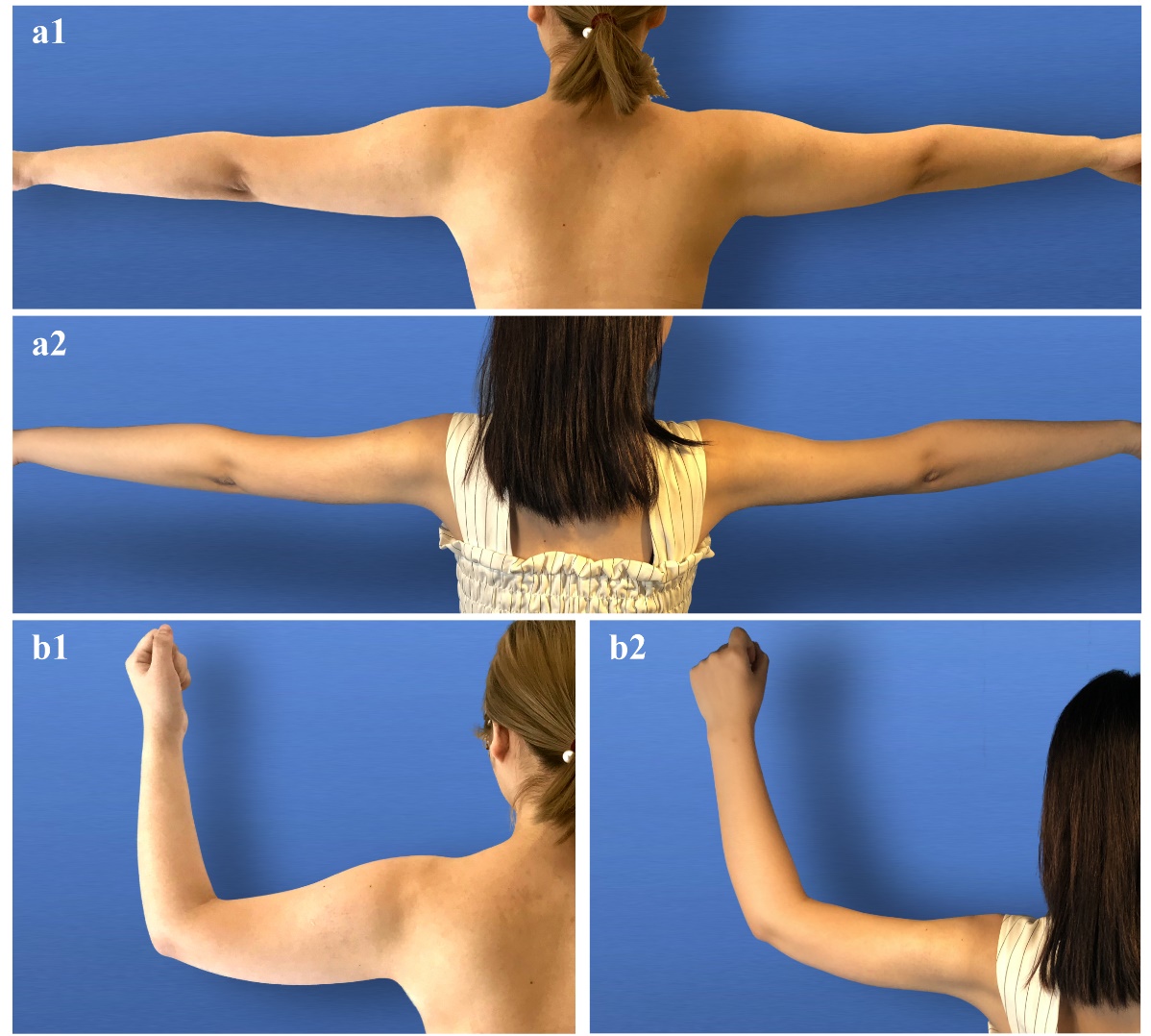


**Figure S5**. The comparison of a type IIIa patient between pre-operation (a1, b1) and three-month post-operation (a2, b2).


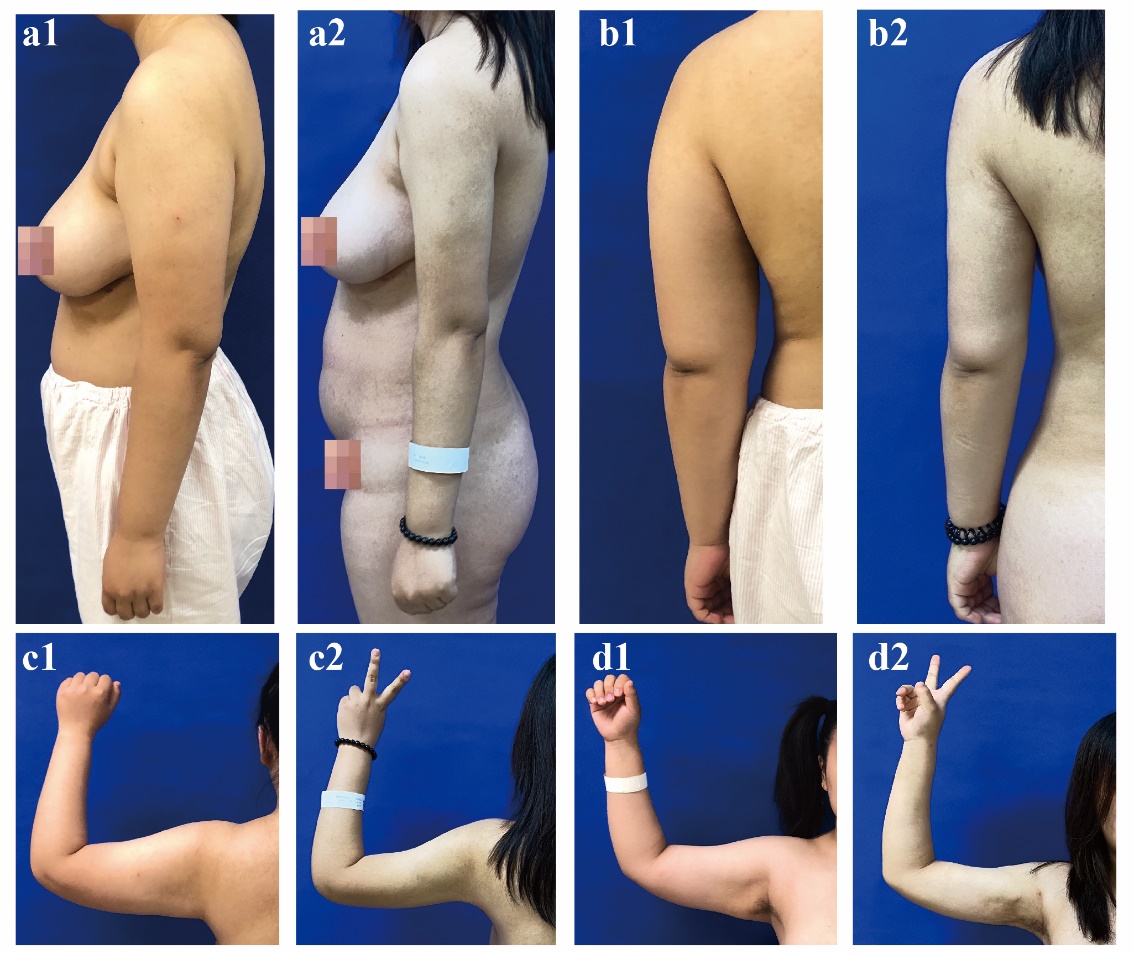


**Figure S6**. The comparison of a type IIIb patient between pre-operation (a1, b1, c1, d1) and three-month post-operation (a2, b2, c2, d2).
